# Rapid Top-Down Control of Behavior Due to Propositional Knowledge in Human Associative Learning

**DOI:** 10.1371/journal.pone.0167115

**Published:** 2016-11-28

**Authors:** Francisco J. López, Rafael Alonso, David Luque

**Affiliations:** 1 Instituto de Investigación Biomédica de Málaga (IBIMA), Málaga, Spain; 2 Universidad de Málaga, Málaga, Spain; 3 School of Psychology, UNSW Australia, Sydney, Australia; Swansea University, UNITED KINGDOM

## Abstract

Propositional and associative processes have been proposed to explain human associative learning. Our main objective in this study was to evaluate whether propositional knowledge may gain control over behavior even under high time-pressure conditions, as suggested by propositional single-process models. In the experiment reported, different groups of participants had to learn a series of cue-outcome relationships on a trial-by-trial basis under different time pressure conditions. Later, a simple verbal instruction indicated that one of the cues had reversed its contingency (*informed* condition). The other cue had also changed its contingency, though in an unanticipated way (*uninformed* condition) whilst other contingencies did not change (*no-change* condition). The results showed that, in the absence of instructions, interference (i.e., *uninformed* vs. *no-change* effect) was greater in the high time than in the low time-pressure group. This result indicates that those responses which were previously relevant are more difficult to inhibit when there is little time to respond. However, time pressure had no detectable effect on the use of the verbal instruction, since an equivalent instruction advantage (i.e., *uninformed* vs. *informed* effect) was obtained in both time pressure groups. These results reveal that propositional knowledge can override those cue-outcome relationships that were learnt trial-by-trial even under conditions of high cognitive demand. This pattern of results is consistent with a propositional single-process model of associative learning.

## Introduction

Since the seminal works by Alloy and Abramson in 1979 [1] and Dickinson, Shanks, and Evenden in 1984 [2], much research interest has been devoted to the study of human contingency learning. The acquisition of knowledge about how predictive cues relate to relevant outcomes serves very well an organism whose aim is adaptive behavior. Knowledge about the contingencies or relationships between cues and outcomes in the world provides individuals with the ability to control the present and predict the future, maximizing the likelihood that they can obtain desired outcomes and avoid non-desired ones [3, 4].

Frequently, a relevant objective of research interest has been to elucidate the process or processes by which the human learner acquires and apply such knowledge (see [5] or [6] for reviews). According to this literature, two main types of model have been proposed, namely, dual-process models [7, 8] and propositional single- process models [5]. Dual-process models assume that two different kinds of processes mediate performance in associative learning tasks, that is, associative and propositional processes, whereas single-process models assume that only the latter play a role in generating participants’ performance. Associative processes assume the operation of learning mechanisms based on association formation algorithms of which Rescorla-Wagner is a representative example [9] (see [10] for a review). The resulting knowledge is stored as a link between the representation of the cue and that of the outcome with "no other property than that of transmitting excitation from one event representation to another” ([11], p. 85). Then, the representation of an outcome is very rapidly retrieved from memory, assuming that activation spreads via associations in the absence of the participant’s cognitive effort or control. On the other hand, propositional processes assume that learning cue-outcome relationships involves a controlled, effortful acquisition of declarative knowledge. This knowledge would be stored in the form of propositions as beliefs about the world. Subsequent behavior would be the result of the application of these cue-outcome beliefs in order to fulfill ongoing goals [12].

Until recently, the majority of associative learning studies had followed a very similar experimental procedure. After giving participants the opportunity to experience a series of actual pairings between cues and outcomes during a learning phase, in a later test phase, participants are asked about the relationships learnt. Although there are various ways in which this test phase may take place, in most of these studies participants are requested to make an explicit verbal judgment on a numerical scale to reflect the magnitude of the relationship detected. But requesting a verbal judgment may be less than ideal if the objective is to evaluate the intervention of associative processes. Usually, participants are provided with sufficient time to give their ratings and thus, goal-directed propositional processes can override responses from any competing associative processes [7, 13].

Data from these experiments have provided a considerable amount of evidence favoring the propositional model [5] and, as a result, defendants of single-process models have seriously questioned the intervention of associative processes [5, 12]. However, more recently, the use of new, more sensitive test procedures that have shown to be effective in revealing associative processes has challenged the propositional account forwarded by the single-process model [8, 14]. These experiments tested in a very rapid manner the knowledge acquired about cue-outcome relationships. The logic for using speeded tests is that, under such conditions of high time-pressure, participants would not have enough time to develop or even retrieve propositions from their memories. Supporting this hypothesis, manipulations that had been used to favor propositional claims have proved ineffective when tests were conducted under high time-pressure. Thus, these authors claim that participants’ performance under such high time-pressure tests was the output of an associative system in which the representation of the outcome is directly activated by the representation of the cue as soon as the cue is perceived, without the implication of any propositional reasoning process. Therefore, following these authors, at least two different systems appear to serve human associative learning, one propositional—responsible for previous results obtained using more standard, slow-paced tests—and another associative, which would be responsible for data obtained in fast-paced tests.

For example, in [14]’s Experiment 4, a dissociation between verbal ratings and speeded performance in a priming test was reported regarding the effects of explicit instructions in the blocking effect (i.e., an effect whereby no predictive value is attributed to a cue—the blocked cue—when this blocked cue is presented together with another cue—the blocking cue—with a higher predictive value [15, 16]). Associative models explain blocking as a consequence of the operation of an associative learning algorithm. Although details could differ from model to model, all associative models assume that blocking is not the product of a deliberative, time-consuming reasoning process. However, these are important features of a propositional account of blocking [17]. In [14]’s Experiments 1–3, blocking was found using a rapid and incidental priming test. Thus, [14] interpreted these results as showing that blocking was produced by an associative mechanism, since a propositional explanation for blocking would require that participants engage in reasoning processes, which are unlikely to operate in an incidental priming test (see [14] for more details). In order to provide stronger evidence for this, their Experiment 4 aimed to discard the operation of propositions during the priming test. In this experiment, the information about the predictive value of the blocking cue was only provided by a verbal instruction that participants could read on the computer screen. A propositional single-process model would predict that both the knowledge acquired on a trial-by-trial basis and that provided by the instructions are stored in memory as propositions. Further, the propositional account predicts that blocking is the result of deductive reasoning based on these propositions. Therefore, blocking would be expected in both the priming and verbal rating tests. However, results have shown that the instructions only produced the blocking effect in the untimed rating test, but not in the priming test under high time-pressure. Given this asymmetry, these authors claimed that at least two systems, one associative and one propositional, underlie participants’ performance on associative learning tasks.

However, data from [14] are not conclusive. As pointed out a number of times, the results from [14] can be viewed as the effect of time pressure over the recovery of propositional knowledge, rather than the operation of two different learning systems [18]. Note that the propositional model of human contingency learning claims that a very fast and uncontrollable retrieval of propositional knowledge is possible [5]. Thus, a propositional explanation of [14]’s Experiment 4 results would still be possible, assuming that those propositions acquired on a trial-by-trial basis were automatically activated in both the priming and the untimed rating tests. According to this, during the incidental priming test, these automatic propositions would control performance directly, since participants had no time (or reason) for overriding them using the propositional knowledge provided by the instructions. However, with more time to think, and when participants were specifically asked about the strength of the target blocked cue-outcome relationship, all sources of information available could be considered, coming to the conclusion that the blocked cue did not have a genuine predictive value, hence the blocking effect shown by the untimed ratings.

However, as appealing as this possibility is, a caveat is necessary here. Recent work has shown that verbal instructions, such as those used in [14], can indeed produce stimulus-response (S-R) mappings that affect behavior in very rapid tests [19–21]. Although these results speak in favor of a propositional single-process model, the dissociations found in [14] (see also [8]) still remain unexplained. In other words, it is not easy to understand why in some cases verbal instructions can rapidly affect behavior [19–21], whereas in others this is not the case [8, 14]. A possible relevant factor to be considered is the source from which those S-R mappings were acquired. For example, in [14]’s experiment, participants acquired *experienced* (i.e., S-R links formed from first-hand experience with cue-outcome parings across trials) as well as *instructional* S-R mappings (i.e., S-R links derived from the verbal instructions provided). Moreover, both mappings led to two incompatible Rs from the same S, one established from the instructions provided, and the other from the direct experience. On the other hand, in most of those studies in which instructional S-R mappings took rapid control of behavior, there were no competing experienced S-R mappings [19–21]. One possibility is that rapid and automatic instructional S-R mappings can be detected in the absence of competing, experienced S-R mappings (as in [19–21]), but they are not strong enough to overrule a preexisting experienced S-R mapping (as in [14]).

In summary, despite its theoretical relevance, it is still not clear whether propositional knowledge derived from verbal instructions can modify preexisting experience-based knowledge when there is little time to think. The present study therefore aimed to fill this gap. To this end, in our experiment participants had to learn relationships between different cues and the correct position of a target outcome in a speeded task, but under a variety of time pressure conditions. In particular, their task was to respond as quickly as possible to the location of the target outcome according to the cue that preceded it. Once these relationships had been learnt, a verbal instruction explicitly stated that the position of the target outcome for one of the cues had changed (i.e., the *informed* cue); following this, the new (reversed) cue-outcome relationship was indicated. Thus, if participants can rapidly retrieve this propositional knowledge and use it to update the S-R mapping acquired during the first learning phase, then their performance should be facilitated with little impact of the time pressure manipulation. This result would favor [5]’s hypothesis concerning a fast intentional retrieval of propositional knowledge and, in addition, it would imply that the retrieved propositional knowledge can effectively reverse the previous experience-based knowledge even under conditions of time pressure. The most parsimonious way to explain this result would be to assume that participants’ performance is driven by a propositional single-process mechanism that is able to operate in both types of S-R mappings, experienced and instructional.

The possible facilitation of the instructions provided about the *informed* cue was evaluated against an *uninformed* cue (i.e., a cue whose cue-outcome contingency had changed but was not referred to by any instructions) as a control condition. What we may call the *Instruction* advantage was calculated as *I = Performance for the uninformed cue* − *Performance for the informed cue*. Finally, the target position of the outcome did not change for the other two remaining cues. These *no-change* cues were used to measure the interference produced by the partial reversal treatment. A *Reversal* interference measure was calculated as *R = Performance for the uninformed cue* − *Performance for the no-change cues*. Single- and dual-process models make different predictions regarding *I*. The dual process model predicts a dissociation in line with [8] and [14]’s results, that is, a smaller instruction advantage (*I*) in the high- than in the low time-pressure condition. As we have mentioned previously, the single-process model predicts no effect of time pressure in *I* scores. On the other hand, for *R*, both models would predict larger scores for the high than the low time-pressure condition. Because the reversal was not signaled by instructions, participants would have had to inhibit the previous correct response to allow for the contingency change. This inhibition process should take time [22], and hence, larger *R* effects are expected in the high than in the low time-pressure condition.

Although measures of *I* and *R* were computed from participants’ performance during the Partial reversal phase, the time-pressure manipulation was implemented from the beginning of the experiment (see Table 1). In order to discard an alternative explanation based on learning differences during the Pre-reversal phase, we also included a group of participants in which the Pre-reversal phase was run under high time-pressure whilst the subsequent Partial reversal phase was run under a low time-pressure condition. Thus, the results obtained during the Partial reversal phase in the low time-pressure condition may not be attributed to the fact that its Pre-reversal phase was also run under a low time-pressure condition, at variance with what occurred during the Pre-reversal phase of the high time-pressure condition.

**Table 1:** Experimental design.

Time pressure	Pre-reversal	Verbal instruction	Partial reversal
High	A – 1 B – 1 C – 2 D – 2	A now goes with 2	A – 2 (informed) B – 1 (no-change) C – 1 (uninformed) D – 2 (no-change)
Low			
High/Low			

Letters stand for cues and numbers for outcomes. The verbal instruction entails a partial reversal of the contingencies programmed in Phase 1. The different types of contingency change are indicated between brackets. An informed change involves a change in the contingency indicated by the verbal instruction whereas an uninformed change involves a change that was not informed. Some of the cue-outcome relationships did not change. An independent group of participants was tested for each of three time pressure conditions programmed: a) high time-pressure, in which both Pre-reversal and Partial reversal phases were conducted under high time-pressure; b) low time-pressure, in which both Pre-reversal and Partial reversal phases were conducted under low time-pressure; and c) high/low time-pressure, in which the Pre-reversal was conducted under high time-pressure but the Partial reversal phase was conducted under a low time-pressure condition.

## Materials and Methods

### Participants and apparatus

A total of 130 Psychology students took part in the experiment in exchange for course credits. The task was programmed using E-Prime 2.0 (Psychology Software Tools, USA). Participants were tested in a quiet room with 10 semi-isolated cubicles equipped with Windows XP PCs (Microsoft, USA). Participants wore headphones at all times throughout the task. Written consent was obtained and the Human Research Ethics Committee of the University of Malaga approved the study.

### Materials

Five geometrical figures were used as stimuli. Four of them (a yellow cross, a light blue half-moon, a blue rhombus, and a green triangle) were used as cues and a green circle was used as the outcome whose position had to be detected on every trial. All cues were presented at the center of the computer screen within an invisible 5 cm x 5 cm square that was maximally occupied by the geometric figures. The green circle had a diameter of 3 cm and could appear at either side (left/right) of the cues at a distance of 2 cm. These different sides (left/right) defined the two outcomes used throughout the task.

### Design

The task was divided in two different learning phases, a Pre-reversal and a Partial reversal phase. In the Pre-reversal phase, four different cue-outcome relationships were programmed (see Table 1 for details). Specifically, two different cues were paired with one of the two outcomes (i.e., a left position of the green circle) and the other two cues with the other outcome (i.e., a right position of the green circle). Seventy-two trials for each of the four cue-outcome relationships were programmed. Once this Pre-reversal phase had finished, participants received a verbal instruction on the computer screen stating that one cue was now paired with the other outcome and were given sufficient time to read and learn the instructions. Following the instructional phase, a Partial reversal-learning phase began. A partial reversal of previous contingencies was programmed for this phase. In particular, one of the cues (i.e., the *informed* cue) was now paired with the other outcome and furthermore, the contingency of another cue was also paired with the alternative outcome (i.e., the *uninformed* cue) whereas the other two cues did not change their relationships with their outcomes (i.e., the *no-change* cues). Thirty-six trials for each of the four cue-outcome relationships were programmed. The two learning phases could take place in three different conditions of time pressure (i.e., Stimulus Onset Asynchrony or SOA). An independent group of participants was tested for each of these three SOA conditions. In the high time-pressure group, on each trial, cues were presented for 250 ms; afterwards, the outcome appeared either to the right or the left of the cue and participants were prompted to give a detection response (left/right) about where the green circle appeared. In the low time-pressure group, the SOA was 1500 ms. In a third group of participants, named High/Low pressure group, the SOA of the Pre-reversal phase was 250 ms and the SOA of the Partial reversal phase was 1500 ms. Participants were randomly assigned to the different time-pressure conditions. A between participants counterbalanced procedure was established so that all the different relationships defined were assigned to each of the geometric figures used. The use of right and left responses as Outcomes 1 and 2 was also counterbalanced between participants (see Table 1).

### Procedure

After reading the general instructions on the computer screen, a practice block of eight trials was presented so that participants could become familiar with the general procedure. All practice trials included the same cue, a different geometrical figure to those described above (a yellow lightning shaped geometrical figure) but of a similar size. On each trial, including practice trials, a 1 cm arm fixation cross appeared in the center of a black silhouette of a 5 cm x 7 cm rectangle against a white background for 500 ms. Then, the cue for that particular trial appeared in the center of the same rectangle for the SOA programmed according to the time pressure condition. Without delay, the outcome appeared either to the left or the right of the cue, outside of the rectangle described, and according to the trial in question. Participants’ responses were registered through the computer keyboard and “z” and “m” keys had to be pressed for a left or right response, respectively, corresponding to where the outcome appeared. Participants used their index fingers from their left and right hand for responses to “z” and “m”, respectively. Though the outcome was present for 500 ms, participants had to make their response during a moving temporal window within those 500 ms. In particular, the temporal window was of 500 ms at the beginning of each learning phase. However, after every correct response (i.e., the correct left/right response for that trial was registered), the temporal window for the next trial diminished in 10 ms and increased by 30 ms after every wrong response (i.e., either the wrong left/right response was given or the response registered was out of the allotted temporal window for that trial). Once the response had been made, participants received either CORRECT/INCORRECT visual feedback in the center of the screen, accompanied by auditory feedback (i.e., a low pitch, 60 dB sound) only in case of an incorrect response. In the event that responses were not registered within the temporal window allotted, a TOO SLOW message was presented, accompanied by the same auditory feedback described for incorrect responses. Corrective feedback was present for 500 ms, after which the fixation cross for the next trial was presented.

Once the first learning stage had finished, a new set of instructions could be read on the computer screen. These instructions stated that the correct response for one of the cues had changed and explicitly stated the cue (i.e., the actual cue appeared) and the new correct response was provided (i.e., a sentence explicitly indicated that the correct answer for the cue was now either left or right). There was no time limit to read these instructions and participants were invited to learn the new contingency. No further cue was mentioned in the instructions. Once participants were ready to resume the task, the Partial reversal phase began. The Partial reversal phase was conducted in exactly the same way as the Pre-reversal phase except for the reversal of the contingencies programmed. Once the Partial reversal phase had finished, the experiment ended and participants left the laboratory once they had all finished the task.

## Results

Responses made outside of the moving temporal window established in the procedure for response registration were referred to as “time outs” and discarded for further analysis. Reaction times (RTs) to incorrect response trials were not considered for further analysis, but incorrect response rates were analyzed (see below). Furthermore, responses from one participant were not included in the analysis as they featured 100% of time out responses during the Partial reversal phase. This high number of discarded responses may be interpreted as indicating lack of attention or commitment during the experimental task. Thus, a total sample of 129 participants was included in the analysis (High = 44, Low = 42, High/Low = 43).

For clarity, we collapsed the data from the two *no-change* cues, as well as the different trials of each trial type within each learning phase. Given that we were interested in measuring the advantage provided by the instructions, either in terms of faster reaction times or lower error rates, both of these measures were integrated in an *Inverse Efficiency Score* (IES; [23]). The IES (expressed in ms) equals the mean RTs divided by the proportion of correct responses, calculated separately for each phase, each condition, and each participant. Lower values on this measure indicate better performance. Once this IES had been obtained, two dependent variables were calculated from the Partial reversal phase data, for each condition and each participant: *(I)nstruction* advantage = IES to the uninformed cue minus IES to the informed cue, and *(R)eversal* interference = IES to the uninformed cue minus IES to no-change cues. Data from each different phase were assessed separately.

**Fig 1:**
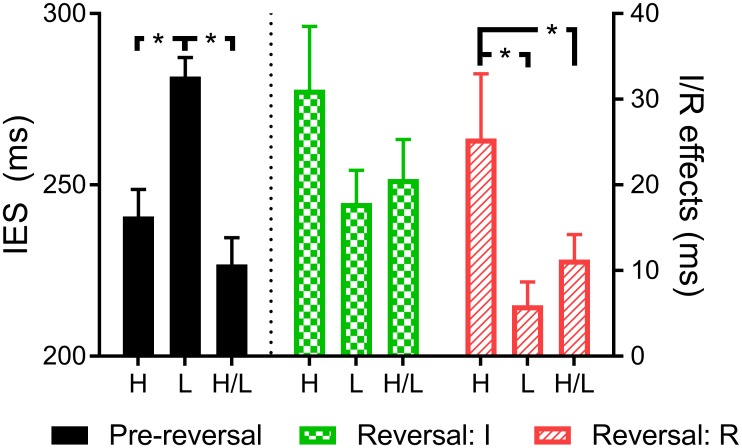
Summary of results. H, L and H/L denote high, low and high/low time-pressure conditions. Error bars represent standard errors of the mean. Black bars on the left side of the figure show mean RTs for correct responses in the Pre-reversal phase (left Y-axis). The rest of the bars show data for the Partial reversal phase; these results are the magnitude of the reversal effects (i.e., right Y-axis scores). Checked bars show the *Instruction* advantage results (*I* effect), while striped bars show the *Reversal* interference results (*R* effect; see main text for a description on how these effects were calculated). Lines and asterisks show significant between-group differences. Notably, while time pressure clearly affected Pre-reversal results and the *R* effect, it did not exert any influence on the *I* effect (all *p*s > .25).

Regarding the Pre-reversal phase, an ANOVA conducted on the IES with *time pressure* (high vs. low vs. high/low) as the only factor revealed a significant effect of this factor, *F* (2, 128) = 15.66, *p* < .001, $\eta_{\text{p}}^{\text{2}}$ = .20. Sidack post-hoc analyses showed that IES in the Low was higher than in the High and the High/Low time-pressure conditions, *p*s < .001. As expected, High and High/Low conditions did not differ, *p* = .427 (see Fig 1). Thus, participants performed worse in the low time-pressure condition.

Regarding the Partial reversal phase, we conducted a one-way MANOVA with *time pressure* (high vs. low vs. high/low) as a factor and *I* and *R* scores as two dependent variables. The result showed that IES-based scores significantly differed in terms of *time pressure*, *F* (4, 250) = 3.23, *p* = .013; Wilk's Λ = 0.904, $\eta_{\text{p}}^{\text{2}}$ = .05. Follow-up tests were therefore conducted. The level of significance was corrected to account for multiple ANOVAs being conducted, using the Bonferroni correction method. Accordingly, in this case, we adopted a significance level of p < .025. Only the *R* score revealed significant differences according to *time pressure*, *F* (2, 126) = 6.41, *p* = .002, $\eta_{\text{p}}^{\text{2}}$ = .09. Tests for the *I* score yielded a non-significant result, *F* (2, 126) = 1.61, *p* = .204, $\eta_{\text{p}}^{\text{2}}$ = .02. See S1 File for equivalent analyses on reaction times and error rates. To further investigate the absence of differences regarding the *I* scores, we performed a Bayesian ANOVA using JASP software [24] and used default priors to estimate the Bayes Factor (BF; [25]). An estimated BF (null/alternative) for the comparison between time pressure conditions yielded an BF_01_ = 3.507 (% of error = 0.044), which suggested that the data were 3.5:1 in favor of the null hypothesis, or rather, 3.5 times more likely to having occurred under a model without including the effect of time pressure, than under a model including this effect.

Post-hoc tests on the *R* score (applying the Sidack adjustment of confidence intervals for multiple comparison) showed that the adverse impact on efficiency of an uninformed change in relations was stronger in the High than in the Low (*p* = .003) and the High/Low conditions (*p* = .019), while these two latter conditions did not differ from each other (*p* = .922). Although the ANOVA and the Bayesian ANOVA did not reveal results supporting the possibility that the *I* score differed across *time pressure* conditions, post-hoc tests were conducted on this variable to confirm that this was the case. None of these three post-hoc tests (Sidack correction applied) yielded a significant result (*p*s > .25). In addition, the *I* effect was greater than 0 in all groups. Three two-tailed one-sample t tests yielded significant results (Bonferroni correction, adopting a statistical significance level of p < .017): High, *t*(43) = 4.18, *p* < .001, *d* = 0.63; Low, *t*(42) = 4.75, *p* < .001, *d* = 0.72; High/Low, *t*(41) = 4.54, *p* < .001, *d* = 0.70.

## Discussion

The main objective of the current experiment was to evaluate whether propositional knowledge may be rapidly retrieved, so that it can override previous S-R mappings acquired through direct experience, regardless of the time pressure with which an associative learning task is carried out. The pattern of results obtained in the partial reversal phase showed that this was precisely the case. A verbal instruction concerning a partial reversal of contingencies was able to produce an instruction advantage even when the cue-outcome relationships were experienced under a high time-pressure condition. In fact, the advantage was equivalent to that obtained when the task was carried out with low time-pressure, and even when pre-reversal contingencies were learnt under high time-pressure (i.e., the high/low condition). The Bayesian analysis revealed relevant evidence for the equivalent results obtained in all three conditions. Specifically, 3.5 times more likely to having occurred under a model without including the effect of time pressure than under a model including this effect. Thus, no matter how pre-reversal contingencies were learnt (either under low or high time-pressure), an equivalent instruction advantage was obtained during the reversal phase. This is, to our knowledge, the first demonstration that verbal instructions can override experience-based knowledge with the same efficiency regardless of time pressure. Overall, these results are consistent with the propositional single-process model [5].

During the partial reversal phase, the results showed a greater reversal interference effect in the high time-pressure than in the two other conditions for the cue whose contingency had suffered an unanticipated change. It is worth noting that this result is consistent with both theoretical approaches, as the high time-pressure condition should impose greater demands on participants’ cognitive resources. One possible way to explain this result is that, given a pre-existent S-R link (whether in a propositional format or not), when the contingencies unexpectedly change for the *uninformed* cue, participants have to re-learn the new S-R contingencies (again, no matter the format). At the beginning of this new learning phase, the two mutually exclusive responses would have been relatively active, and some effortful inhibition process should have taken place to solve the conflict. It is expected from both single- and dual-process models that this inhibition process demands time to operate [22] and thus, it is more efficiently carried out under low than high time-pressure conditions.

In relation to the results obtained during the pre-reversal phase, participants’ inverse efficiency scores (IES) were greater for the low than for the high time-pressure conditions. This result very likely indicates that participants were allocating more cognitive resources for these high-pressure conditions, due to the greater difficulty, and thus, their performance was more efficient when required to do the more demanding task. Again, this result may be understood in terms of both theoretical accounts.

Therefore, in general, it appears that there is nothing in the current pattern of results that goes beyond the predictions of propositional single-process models. The main result—the instruction advantage shown under high time-pressure—may be interpreted as showing that propositional knowledge was effectively retrieved to guide participants’ performance during the reversal phase. In other words, this result provides empirical support for [5]’s suggestion of a very fast recollection of propositional knowledge—a notion that until now had received little empirical support.

De Houwer and colleagues [26, 27] have provided evidence in favor of the involvement of propositional knowledge in evaluative conditioning and causal learning using the Implicit Association Test (IAT) [28]. The IAT provides a clear advantage over more standard paradigms (e.g., based on the use of verbal judgments) to favor faster responses, which, in principle, should be more sensitive to detecting the operation of associative processes. However, participants’ performance, as measured by the IAT, has not been collected under time pressure in those experiments (i.e., participants’ responses were not discarded as errors when recorded outside of an allotted temporal window). Thus, our experimental procedure may be regarded as a more stringent test for revealing a fast activation of propositional knowledge. Even more compelling are the results reported by Mertens and colleagues [29, 30] using a fear-conditioning paradigm. In their studies, participants first learned that certain conditioned stimuli were associated with shocks, while others were safe. Following this, a verbal instruction stated that the contingencies had been reversed in subsequent trials. Interestingly, following the verbal instruction, fear potentiated startle responses were reversed. Despite relevant procedural differences between these results and those reported here (e.g., the use of aversive shocks in their procedure), there are relevant analogies between them. Firstly, in both studies, participants’ responses were very rapid (i.e., potentiated startle responses and responses under high time-pressure). Secondly, in both studies, these instruction-based rapid responses could actually override pre-existing experience-based S-R mappings. Thus, both our results and those of [29, 30] may be regarded as providing convergent evidence for a rapid control of behavior due to instructions, even when previous experience-based S-R mappings had to be overruled.

As we have noted in the introduction, previous experiments using fast-paced tests have shown results consistent with a dual-process model. It is unlikely that these findings could be attributed to their use of stricter time-pressure conditions than those used in our procedure. In particular, it should be noted that the time pressure used in our task—a SOA of 250 ms.—is similar to that used in those studies showing evidence in favor of a dual-process account (ranging from 200 to 350 ms.; [8, 14, 31, 32]). In other words, a SOA that was able to preclude a fast retrieval of propositional knowledge to control performance in these experiments, revealed dissociative results. Why, then, has a similar SOA produced results consistent with a propositional single-process account in our experiment? A tentative answer to this question would point to the simplicity of the reversal instruction provided (i.e., cue X predicts outcome Y). This simplest form of instruction is likely to have facilitated the operation of controlled processes to retrieve propositional knowledge during the reversal phase, overriding experienced-based S-R mappings. This facilitation took place even under similar time pressure conditions to those used in previous studies, but in those previous studies more complex forms of propositional knowledge had to be retrieved (in a blocking situation, for example, more complex inferences are involved: the true predictive value of a target cue is computed by discounting the predictive value of other cues that had accompanied this target cue).

This leads us to the important issue of the range of parameters that characterize the detection of single or dual-process mechanisms as an account of participants’ performance. For example, until now a SOA of 250 ms. together with other task features (see [14] for more details) was assumed to preclude the fast retrieval of propositional knowledge, but our results have shown that this is not always the case. Thus, a single-process account has shown to be sufficient to understand participants’ performance even under high time-pressure when simple propositional knowledge needs to be retrieved. It is possible that this time-pressure will not allow the use of more complex propositional knowledge or the retrieval of propositional knowledge may prematurely end, which in some cases might lead to an output similar to that predicted by associative learning models [5].

Though our research interest has focused on the theoretical debate regarding the nature of processes underlying human associative learning, it should be noted that the implications of our results go beyond this debate. For example, despite classical theoretical controversies concerning the issue of knowledge format (e.g., whether propositions may lead to associations or vice versa under certain circumstances [33, 34]) our results have shown that top-down influences may effectively control performance, even under conditions in which little time may be devoted to thinking or reflection. In other words, regardless of the specific format of knowledge representation, our results have revealed that knowledge derived from verbal instructions may alter previous experience-based S-R mappings, which shows, in turn, that there is a common ground in which knowledge from different sources can be considered for behavioral adaptation, even under high time-pressure conditions. Moreover, this is an important and novel result. To our understanding, there is an important difference between previous studies based on IAT (e.g., [26–28]) or potentiated startle responses (e.g., [29, 30]) and the results reported here. Whilst these previous demonstrations of the operation of propositional knowledge in performance were detected without effortful control on behalf of the participants, our participants were instructed to use the verbal information provided in order to adapt to the new contingencies. Thus, we are showing that instructions can have a very rapid and effective intentional effect on behavior. This result in itself has relevance from an applied point of view. In particular, voluntary control of performance may have consequences when dealing with some forms of abnormal behavior in which rapid, unwanted responses are elicited by specific stimuli such as, for example in *habit* behavior as opposed to *goal-directed* behavior (e.g., [35]). The results suggest the possibility that such forms of rapid involuntary responses may be counteracted very quickly by more appropriate and voluntary responses elicited by specific stimuli. Knowledge derived from instructions, such as those provided in psychotherapy, may serve to activate these more appropriate responses as soon as specific eliciting stimuli are detected. In fact, this is precisely the possibility that is suggested by our findings; a more appropriate, now correct response, has overridden an old response elicited by a target stimulus. Only future research will be able to determine whether this potential applied benefit of our results can be effectively put into clinical practice.

## Supporting Information

S1 File (DOCX)
