# Supplementary material for: Rapid Top-Down Control of Behavior Due to Propositional Knowledge in Human Associative Learning
Source: PLoS One. 2016 Nov 28;11(11):e0167115. doi: 10.1371/journal.pone.0167115 (PMC5125669; doi:10.1371/journal.pone.0167115)
Supplement: S1 File — (DOCX) [file pone.0167115.s001.docx]

**Supporting Information**

[López et al., Rapid top-down control of behavior due to propositional knowledge in human associative learning, *Plos One*]

**Analyses of Reactions Times and Error rates**

The same analysis was performed as in the IES (see main text), that is, one-way MANOVA with *time pressure* (high vs. low vs. high/low) as a factor and *I* and *R* scores as two dependent variables. We corrected alpha for the follow-up tests (significance criterion set at p < .025)

**Reaction Times**

**MANOVA**. *F* (4, 250) = .96, *p* = .428; Wilk's Λ = 0.970, **$\text{η}_{\text{p}}^{2}$**= 0.02.

**Follow-up tests.** *R* score: *F* (2, 126) = 1.61, *p* = .204, **$\text{η}_{\text{p}}^{2}$** = 0.03. *I* score: *F* (2, 126) = 0.09, *p* = .909, **$\text{η}_{\text{p}}^{2}$** < 0.01.

**Error Rates**

**MANOVA:** *F* (4, 250) = 6.17, *p* < .001; Wilk's Λ = 0.828, **$\text{η}_{\text{p}}^{2}$** = 0.09.

**Follow-up tests.** *R* score: *F* (2, 126) = 12.81, *p* < .001, **$\text{η}_{\text{p}}^{2}$**= 0.17. (High, *M* = .051, *SEM* = 0.007; High/Low, *M* = .003, *SEM* = 0.008; Low, *M* = .007 *SEM* = 0.008. Sydack corrected post-tests: High vs High/Low, *p* < .001; High vs Low, *p* < .001; Low vs High/Low, *p* = .980). *I* score: *F* (2, 126) = 3.27, *p* = .041, **$\text{η}_{\text{p}}^{2}$** = 0.05.

Therefore, the only significant effect of time pressure was for Accuracy, specifically for the *R* score and not for the *I* score, in line with the results obtained when the IES measure was used.
